# Effects of Microwave on Mortality and Detection Efficiency of Three Stored Grain Insect Adults in Stored Paddy, and on Grain Quality

**DOI:** 10.3390/insects17010067

**Published:** 2026-01-06

**Authors:** Shiyuan Miao, Yiting Zhou, Suisui Wang, Zhipeng Yang, Adrien Guverinoma, Yaru Zhao, Yujie Lu

**Affiliations:** 1 School of Grain Science and Technology, Jiangsu University of Science and Technology, Zhenjiang 212003, China; 251112101105@stu.just.edu.cn (Y.Z.); ms.susan.wang@outlook.com (S.W.); 241212101116@stu.just.edu.cn (Z.Y.); 250511800008@stu.just.edu.cn (A.G.); zhaoyaru@just.edu.cn (Y.Z.); 2 School of Agricultural Science, Murdoch University, 90 South Street, Murdoch, WA 6150, Australia

**Keywords:** *Sitophilus oryzae*, *Tribolium castaneum*, *Oryzaephilus surinamensis*, lethal effects, recovery percentage, storage stability of paddy

## Abstract

Microwave heating has been widely used for disinfestation in the food industry due to its selective heating. However, research on the effects of microwave heating on stored product insects is still relatively limited, which has restricted its broader application in grain pest control storage. Therefore, this study evaluated the lethal effects of different microwave powers and exposure times on three major pests in paddy and investigated the impact of microwave treatment on improving adult detection efficiency, intending to develop a rapid and efficient detection method for stored grain insects. The results showed that the mortality of *Sitophilus oryzae*, *Tribolium castaneum*, and *Oryzaephilus surinamensis* increased with the increase in microwave power and exposure time. Specifically, 100% mortality was achieved for both *S. oryzae* and *T. castaneum* at 700 W for 60 s exposure. However, higher power levels and longer exposure durations exacerbated the non-uniformity of grain temperature distribution and adversely affected the germination rate. In addition, microwave treatment at 350 W, 490 W, and 700 W significantly reduced fungal load in paddy. The moisture content and water activity of rice decreased with the increase in microwave power and exposure time, while the percentage of grain breakage remained largely unaffected. These findings indicated that microwave treatment can effectively control insects and fungi without significantly altering the main physical properties of paddy. Notably, microwave treatment with short exposure durations (20–30 s) at all three power levels is conducive to increasing the recovery percentage of *S. oryzae* adults, while microwave treatment at low power (350 W) with exposure durations of 25–40 s helps improve that of *T. castaneum*. Accordingly, microwave heating is not only a promising strategy for protecting stored grains but also has potential for development as a rapid detection method for specific insect pests.

## Simple Summary

Paddy (*Oryza sativa* L.) is one of the most important and high-yielding grains in the world. However, during storage, pests are often hidden within the protective layer formed by the husk, making them difficult to detect through conventional methods. *Sitophilus oryzae*, *Tribolium castaneum*, and *Oryzaephilus surinamensis* are three major pests that infest stored paddy. The current detection methods for these pests largely rely on traditional methods, which are time-consuming, labor-intensive, and are heavily based on manual operation. This study evaluated the potential of microwave treatment for controlling these pests and improving their adult’s detection in paddy samples. The results showed that as microwave power and exposure time increased, the survival rates of all three insect species and the fungal load in paddy decreased. Furthermore, moderate microwave treatment improved key physical properties of paddy, such as moisture content, water activity, and grain breakage percentage, thereby enhancing its storage stability. These findings suggest the possibility of developing an effective method for controlling insects and fungi to extend the storage quality of paddy. Additionally, short-duration and low-power microwave treatment significantly increased the recovery percentages of *S. oryzae* and *T. castaneum*, indicating the potential of this technique as a simple, rapid, and efficient method for detecting stored product insects in grain samples.

## 1. Introduction

Paddy (*Oryza sativa* L.) is a major cereal crop globally, both in terms of production and consumption [1]. It is a critical source of carbohydrates, essential amino acids, and micronutrients such as B vitamins [2], which provide vital energy and nutrition for humans [3]. To ensure year-round availability, harvested paddy is often stored for extended periods before processing and consumption [4]. However, post-harvest storage losses pose a significant threat to grain preservation and food security. Specifically, the paddy is susceptible to infestations from different pests during storage, which can result in substantial quantitative and qualitative damage. The pests in stored grains severely threaten the safety of rice grains primarily through the following ways: directly boring into intact grains, contaminating the grain bulk by secreted toxins, excrements, exuviae, etc., and infesting damaged rice kernels. The rice weevil *Sitophilus oryzae* (L.) is a primary stored grain insect pest in paddy [5]. This species can infest and reproduce within intact cereal grains, making it particularly difficult to control [6]. The red flour beetle *Tribolium castaneum* (Herbst) is a major secondary stored pest of wheat and rice mills, processing facilities, and food warehouses [7]. Adults can secrete benzoquinone derivatives—carcinogenic substances that lead to the deterioration of grain quality and seriously endanger the quality of processed grains [8]. The saw-toothed grain beetle *Oryzaephilus surinamensis* (L.) is also an important secondary stored grain pest, feeding on damaged rice kernels and processed cereal products [9]. Its flat morphology and crevice dwelling habits further increase the difficulty of prevention and control. Commonly, chemical insecticides such as fumigants and residual grain protectants are used for controlling stored product pests [10,11]. However, long-term and large-scale reliance on chemical insecticides has been widely reported to facilitate the development of pest resistance [12]. In addition, their application has also raised concerns about potential environmental pollution, residue, and food safety risks [13]. Consequently, there is an urgent need to develop efficient, safe, and environmentally friendly alternative strategies for controlling insects infesting stored grains and prolonging the storage quality of grains.

One feasible alternative solution that has been studied is the use of microwave technology for grain insecticidal treatment before storage, mainly based on its potential of selective heating, pollution-free operation, and quality preservation [14,15,16]. However, to achieve wider application and promotion, it is still necessary to carefully consider its energy efficiency as well as safety prevention and control measures against metal impurities. Multiple studies have demonstrated that microwave heating has been verified to be effective in killing stored grain pests in wheat [14,17], barley [18], corn [19], rice [20], and dates [21] through laboratory and small-scale trials. However, systematic research on the insecticidal efficacy of hidden insect pests in paddy, a crop with a special hull structure, following microwave treatment, remains relatively scarce.

Microwave heating is based on electromagnetic radiation at specific frequencies interacting with dielectric materials, inducing the rapid movement and rotation of ions and polar molecules within the exposed material. The consequent intermolecular friction converts electromagnetic energy into thermal energy, and the temperature in the treated material increases [4,22,23]. Since microwave heating relies on the dielectric properties of the material, it is possible to selectively heat insects in grain because of their higher water content [20]. Microwave treatment induces high-temperature short-duration heating, which may not damage the nutritional value of grains as much as prolonged convective drying heat treatments [24,25]. It was reported that microwave heating at low power can be applied as an effective treatment for improving the physicochemical and nutritional properties of sorghum grain [26]. Moreover, recent research has found that microwave treatment at 500 W for 120 s does not cause significant effect on the proximate composition, thermal properties, FTIR signature spectra, cooking properties, and sensory properties of milled rice [20]. However, the thermal effect of microwave treatment may also negatively impact grain quality. For instance, microwave treatment at 400 W for 56 s reduced the α-amylase, saccharifying power, soluble protein, viscosity, and density of the barley malt [18]. Moreover, the germination capacity of the barley also decreased with power level and exposure time increasing [18]. Similarly, microwave heating (300 W) that raised the temperature of corn grains to 50, 55, and 60 °C significantly reduced their germination rate [25]. Verma et al. found that the microwave energy and time used considerably impacted the physicochemical properties of raw and cooked rice [24]. Therefore, to improve the efficiency of microwave disinfestation in stored grains, it is necessary to systematically evaluate the effect of microwave energy and exposure time on grain quality.

In addition to controlling stored-product pests, microwave heating holds significant potential for detecting insect pests in stored grains. The manual sampling and screening methods are commonly used to estimate insect numbers and the level of infestation in grain handling facilities [27,28]. However, these conventional methods fail to detect internal grain infestation and incompletely separate all external insects from the samples. To address these limitations, Jian et al. (2015) developed a new method that combined microwave heating with sieving to force insects out of grain kernels, enabling rapid quantification of *Cryptolestes ferrugineus* (Stephens) in stored wheat [29]. Moreover, their comparative analysis further demonstrated that the microwave heating had the highest recovery percentage of introduced insects from infested wheat among the methods tested: Berlese funnel, shaking grain on a metal sieve, and shaking grain on an acrylic device [27]. Nevertheless, while the efficacy of microwave heating in expelling insects from wheat has been established, its applicability to paddy with husk remains uncertain. The lemma-palea structure of rice husks serves as a physical barrier that protects nutrients from loss or destruction due to internal and external factors during storage [30]. Its complexity likely increases the difficulty of detecting storage pests. Therefore, optimizing microwave treatment parameters to improve detection efficiency in stored paddy is of practical significance for developing rapid and efficient monitoring technologies for stored grain pests, especially for routine detection and early warning in grain depots.

Given the importance of microwave heating for insect control and detection in stored grains, this study evaluates the efficacy of various microwave exposure regimens on three common insect species infesting paddy through small-scale experiments. While Jian et al. (2016) [27] demonstrated that microwave treatment could improve the detection efficiency of stored product insects, their methods involved relatively long exposure times (150 s). It has been reported that the long application time of microwave heating may adversely affect grain quality, such as by reducing the viscosity and antioxidant activity of sorghum grains [31]. Therefore, to enable the safe and efficient application of microwave heating in insect detection, further research is needed to optimize the treatment conditions. Therefore, considering the potential negative effects of microwave treatment on grain quality, we have determined the physical properties of paddy at specific parameters optimized for insect control after microwave heating. This research seeks to provide insights into the potential of microwave heating as a reliable and efficient tool for stored grain disinfestation and pest detection in paddy storage.

## 2. Materials and Methods

### 2.1. Insects

The insects of *S. oryzae* and *O. surinamensis* species were collected from a grain depot in Zhenjiang. The GA-1 strain of *T. castaneum* was obtained from Henan University of Technology and cultivated over 20 generations by the Laboratory of Stored Grain Insects at Jiangsu University of Science and Technology. The rice weevil was reared on brown rice with a moisture content of 14% and cultured in an incubator at 30 ± 2 °C, 65 ± 5% RH [6]. The red flour beetles *T. castaneum* were fed with whole wheat flour mixed with the Brewer’s yeast flour (19:1, weight/weight) and kept in an incubator at 30 ± 1 °C and relative humidity of 40 ± 5% RH [32]. The saw-toothed grain beetle *O. surinamensis* was reared at 25 ± 1 °C and 65 ± 5% RH under complete darkness with the diet of a mixture of whole wheat flour: rolled oats: yeast: crushed wheat = 9:3:3:1 (weight/weight) [9]. The paddy (*Oryza sativa* subsp. Geng/japonica) (moisture content 13.5%) used in this study was purchased from a grain depot in Zhenjiang.

### 2.2. Microwave Treatment Setup

A domestic microwave oven (Model: Midea M1-L213B, Shanghai, China, 2450 MHz, internal cavity dimensions: 27.5 cm × 25.5 cm × 17.5 cm) was used for all treatments. The paddy samples were prepared by placing 200 g of clean, insect-free paddy (previously frozen at −20 °C and sieved to remove any debris) into a polypropylene container (volume: 1.2 L, diameter: 14.5 cm, height: 7.5 cm). Subsequently, 50 unsexed adults of each insect species (*S. oryzae*, *T. castaneum*, and *O. surinamensis*) were introduced into the container and gently mixed with the grains to achieve a uniform distribution. The sample formed a consistent layer approximately 3.5 cm thick at the bottom of the container. The samples were subjected to microwave heating at three different output power levels: 350 W, 490 W, and 700 W. At each power level, six exposure durations were tested: 10, 20, 30, 40, 50, and 60 s. Each combination of power and exposure time constituted one treatment. A separate batch of paddy and insects, prepared identically but not subjected to microwave irradiation, served as the control (0 s). All treatments, including the control, were replicated three times using independent samples. Immediately after each microwave treatment, the surface temperature distribution of the grain mass was captured using a UTI120S infrared thermal imager (UNI-T, Dongguan, China), as detailed in Section 2.4.

### 2.3. Mortality of Three Stored Product Insects in Paddy After Microwave Exposure

Paddy samples were prepared by combining a batch of 200 g of clean paddy (pre-treated by freezing at −20 °C to kill all living insects and subsequently cleared of insect bodies) with 50 adults of each of the three insect species. These samples were placed into polypropylene containers (volume 1.2 L, diameter 14.5 cm, height 7.5 cm) and subjected to microwave heating under different combinations of output power (350, 490, and 700 W) and exposure time (10, 20, 30, 40, 50, and 60 s). After microwave treatment, the samples were immediately placed in a BLH-2810 Electromagnetic Sieve Shaker with the apertures of 2.0 mm (Bethlehem, Taizhou, China) to screen and extract the insects. Specifically, the samples were rotated forward for 1 min and then reversed for 1 min at 110–120 r/min to separate insects from the paddy samples. Afterwards, both surviving and dead individuals on the bottom sieve were collected and counted. Paddy samples that were not subjected to microwave treatment served as the control group. Three replicates were performed for each microwave treatment condition. Adult insects were considered dead if they showed no response to gentle prodding with a brush applied to the abdomen or antennae observed under an Olympus SZX16 stereomicroscope (Olympus, Tokyo, Japan). The immediate mortality percentage of each species was calculated as the number of dead adults in each trial divided by the initial number of live adults tested [20].

To investigate the delayed insecticidal efficacy of microwave heating, all survived adults of each treatment were transferred into glass bottles, each containing 2 g of species-specific diet. Subsequently, these bottles were placed in the incubators under the temperature and relative humidity conditions described in Section 2.1. And 72 h later, the delayed mortality of each treatment was observed and counted. The delayed mortality percentage of each species was calculated as the number of total dead adults in each trial divided by the initial number of adults tested [33].

### 2.4. Temperature Monitoring of Paddy Samples After Microwave Exposure

To monitor the effect of microwave exposure on surface temperature of each sample, the infrared image was conducted immediately by an UTI120S infrared thermal imager (UNI-T, Dongguan, China). The average temperature on the surface of the grain bulk was calculated as the mean value of temperatures measured at five points: four diagonal points located at the external positions of the circular area on the grain surface and one central point within this area. The maximum and average surface temperature were simultaneously recorded for each sample [14]. Each microwave treatment was performed in triplicate, with paddy samples not suffering microwave exposure serving as the control (0 s under microwave treatment). It should be noted that this method captured only the surface temperature distribution; internal grain temperatures may differ and were not directly measured in this study.

### 2.5. Effects of Microwave Treatment on the Physical Properties of Paddy Grains

After separating insects from paddy samples subjected to various microwave exposure conditions, several physical properties of paddy grains were evaluated: moisture content, water activity, grain breakage percentage, and germination rate. The moisture content was determined by the hot air oven method [21]. Approximately 10 g of thoroughly ground paddy seeds were randomly sampled, placed in an aluminum moisture box, and dried in a pre-heated oven (Yiheng, Shanghai, China) at a temperature of 140 ± 5 °C for at least 40 min. The moisture content percent was calculated based on the weight loss relative to the initial weight [34]. The water activity (Aw) was determined using a water activity meter (HD-3B, Wuxi, China), which has an accuracy range of 0.03–0.99 Aw. Grain breakage percentage was derived as the ratio of the weight of broken grains after microwave treatment to the initial total weight. Meanwhile, the germination of paddy seeds was performed using the Between Papers method [34]. Specifically, fifty seeds were placed between two moist filter papers in a small fruit preservation box, with 5.5 mL of distilled water added. The boxes were kept at 25 ± 1 °C and 65 ± 5% RH. After seven days, germinated seeds were counted, and the germination rates were calculated accordingly [34].

### 2.6. Determination of Total Fungal Content

Untreated and treated paddy samples were collected separately and placed in sterilized high-density polyethylene sample boxes for fungal quantification. Approximately 1 g of each sample was weighed, and homogenized in 10 mL of 0.1% peptone water solution to obtain an initial dilution of 1:10. Subsequently, 100 µL of the dilution was spread onto potato dextrose agar (PDA) plates. The inoculated plates were then incubated at 28 °C for five days in a microbial incubator to facilitate fungal growth. The colony count was expressed as colony-forming units per gram (CFU/g) of sample [26].

### 2.7. Recovery Percentage of Adults of Three Pest Insect Species in Stored Paddy Samples

**Figure 1:**
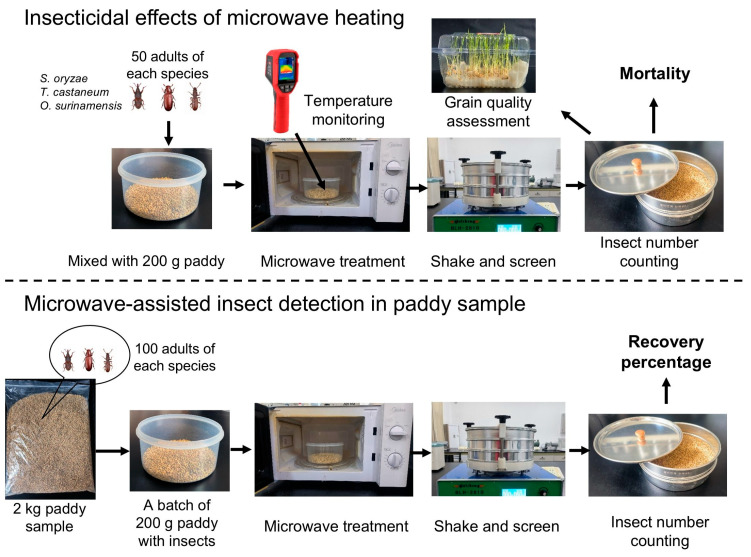
Schematic diagram illustrating the study workflow. The key components of the experimental process include evaluation of immediate and delayed mortality of three stored product insect species in paddy under different microwave power and exposure time combinations, measurement of grain temperature and quality parameters following microwave exposure, and assessment of insect recovery percentage using a microwave-assisted sieving method.

Two kilograms of pretreated clean paddy with a moisture content of 13.5% were transferred into a plastic Ziplock bag and stored for at least 24 h at room temperature. Then, 100 adults of each insect species (*S. oryzae*, *T. castaneum*, and *O. surinamensis*) were introduced into the bag, which was maintained at room temperature for 5 d. Subsequently, 200 g samples of the infested paddy were placed separately into a polyethylene container and subjected to microwave treatments with various combinations of output power (350, 490, and 700 W) and exposure time (20, 25, 30, 35, and 40 s). To assess the detection efficiency, a batch of 200 g samples were randomly taken from each container for analysis. Untreated samples served as the control group. Each experimental treatment included three replicates. The insects in the paddy samples were screened and extracted using the method described in Section 2.2. Each bag of paddy sample was tested 10 times, and the cumulative number of individuals of each species collected from each screening was recorded as the actual number detected. The recovery percentage for each sample was defined as the ratio of total number of collected individuals to the total number of insects released [29]. The workflow of this study is illustrated in Figure 1.

### 2.8. Statistical Analysis

All statistical analyses were performed using SPSS (version 20.0, IBM Corp., Armonk, NY, USA), and figures were generated using GraphPad Prism (version 8.3, GraphPad Software, Inc., San Diego, CA, USA). Data are presented as mean ± standard error of the mean (SEM). Prior to conducting parametric tests, the assumption of homogeneity of variances across treatment groups was formally assessed. For all datasets pertaining to insect mortality and recovery percentage, the Brown–Forsythe test was employed due to its robustness to deviations from normality. The results confirmed that the assumption of homoscedasticity was not violated for the main comparisons (all *p* > 0.05). Consequently, a one-way analysis of variance (ANOVA) was deemed appropriate. Where the ANOVA indicated a significant main effect (*p* < 0.05), Tukey’s honestly significant difference (HSD) post hoc test was applied for multiple comparisons between treatment levels. The use of parametric tests (one-way ANOVA) for proportional data (mortality and recovery rates) was justified as the primary assumptions were met. The homogeneity of variances across all treatment groups was confirmed using the Brown–Forsythe test, and residual diagnostics did not indicate severe deviations from normality. Continuous data on grain temperature, moisture content, water activity, grain breakage percentage, germination rate, and fungal load (log CFU/g) were also analyzed using one-way ANOVA followed by Tukey’s HSD test, as these data met the assumptions of normality and homoscedasticity as verified by the Shapiro–Wilk and Brown–Forsythe tests, respectively.

## 3. Results

### 3.1. Mortality of Three Stored Product Insects in Paddy After Microwave Treatment

To evaluate the insecticidal efficacy of microwave treatment on three stored product insects in grain media, insects in paddy were exposed to microwave heating. The immediate mortality of the three pest species was measured after treatment. As shown in Table 1, a significant increase in the immediate mortality of *S. oryzae* with increasing power was observed at microwave exposure times of 50 and 60 s. At power levels of 350 W and 490 W with a 50 s exposure, the mortality rates were 72.00% and 80.67%, respectively, whereas 50 s exposure at a power level of 700 W resulted in 89.33% mortality rates. Particularly, the mortality rate dramatically increased to 100% at a power level of 700 W for 60 s. In addition, extending the microwave treatment time from 10 s to 60 s significantly increased the immediate mortality. At power levels of 350, 490, and 700 W with a 30 s exposure time, the mortality rates were 36.00%, 54.00%, and 56.00%, respectively, while those with a 40 s exposure time resulted in 62.00%, 65.33%, and 72.00% mortality rates, respectively. Similar phenomena were also observed in the *T. castaneum* (Table 1). In addition, the immediate mortality of *O. surinamensis* increased with increasing microwave power and exposure time. Despite this overall trend, no significant difference was observed among power levels of 350 W, 490 W, and 700 W at comparable exposure times. These results indicate a strong correlation between increasing exposure time and an increased immediate mortality of three stored insects in paddy.

To evaluate the delayed lethal effect of microwave irradiation on pests in paddy, the surviving adults after treatment were transferred to constant temperature and humidity incubators for further observation. The delayed mortality in each group was systematically recorded after 72 h. Results showed no significant differences between delayed and immediate mortality rates for all three pest species. For instance, in the 700 W/40 s treatment group, the immediate mortality rate of *S. oryzae* was 72.00%, and it only fluctuated slightly to 73.33% after 72 h (Table 1). A similar pattern was observed in the other two pest species (Table 1). These findings suggested that microwave heating did not induce a significant delayed lethal effect on pests infesting paddy grains.

### 3.2. Effect of Microwave Treatment on the Temperature of Paddy Samples

**Figure 2:**
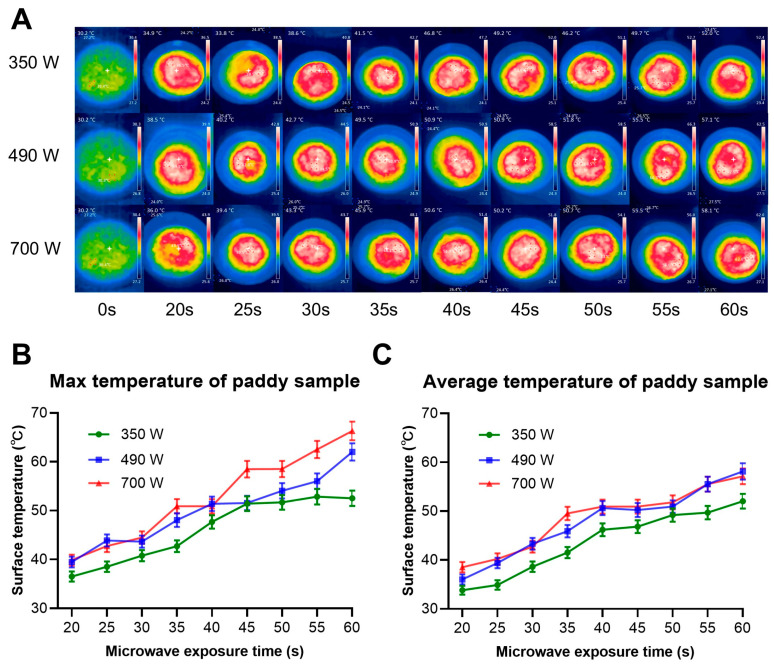
Effect of microwave treatment on the temperature of paddy samples. (**A**) Infrared images of paddy samples exposed to different power levels and durations. (**B**) The effect of microwave power and exposure duration on the maximum surface temperature. (**C**) The effect of microwave power and exposure duration on the average surface temperature. Data were shown as mean ± SEM of three replicates.

Figure 2A illustrates the influence of different microwave power levels and exposure durations on the maximum temperature of 200 g paddy samples. The temperature data was directly obtained from infrared images captured immediately after microwave treatment. The color temperature distribution in Figure 2A indicates uneven heating, with higher temperatures observed in the central zone of the sample than in the outer ones. This is a characteristic attributed to the geometry of the microwave radiator and the distribution of the microwave field, as also reported [35]. At a power level of 350 W, the maximum temperature increased with prolonged exposure time and gradually trended to a plateau value (Figure 2B), likely reflecting a thermal equilibrium between the heat absorbed by the sample (limited by the microwave power) and the heat dissipated through radiation and convection [14]. In contrast, at power levels of 490 W and 700 W, the maximum temperature continued to rise with extended exposure time, showing a clear divergence from the trend observed at 350 W. This difference may be due to the faster rate of microwave energy transfer to the sample at higher power levels, leading to more rapid temperature increase and reaching a higher level in a shorter period. Furthermore, the average surface temperature of the sample was determined by calculating the mean value of the central point and four external points. At a power level of 350 W, the average temperature progressively increased with prolonged exposure time. At power levels of 490 W and 700 W, the average temperature exhibited a phased pattern: it increased with exposure time during the initial 40 s, stabilized with no obvious change between 40 and 50 s, and resumed an upward trend as the exposure time extended from 50 to 60 s (Figure 2C). Further analysis revealed that the difference between the maximum surface temperature and the average temperature increased with extended exposure time at 700 W. For instance, at a power level of 700 W and 30 s of exposure time, the maximum and average surface temperatures were 44.5 °C and 42.7 °C, respectively. After 60 s of exposure at the same power level, these values increased to 66.3 °C and 57.1 °C, respectively. These results indicate that higher power and longer duration do not necessarily lead to stronger heating effects and may instead exacerbate the non-uniformity of temperature distribution.

### 3.3. Effect of Microwave Treatment on the Physical Properties of Paddy

**Figure 3:**
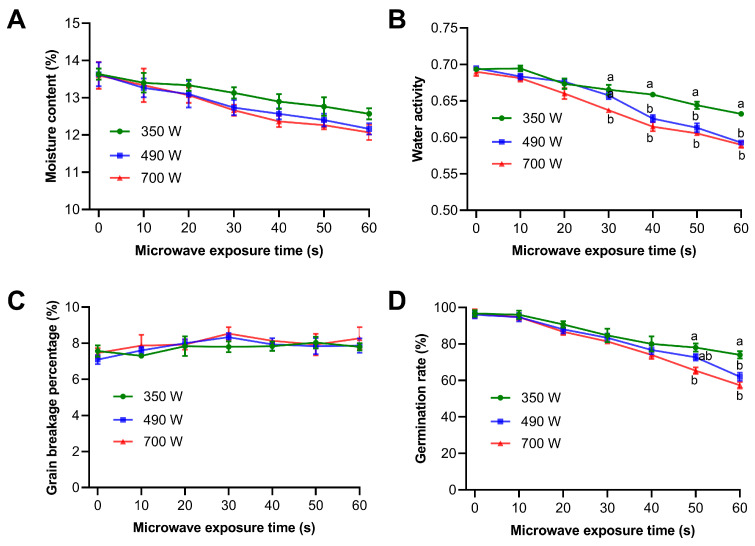
Effect of microwave treatment on the physical properties of paddy. The moisture content (**A**), water activity (**B**), grain breakage percentage (**C**) and germination rate (**D**) of paddy after microwave exposure. Data were shown as mean ± SEM. The lowercase letters above the error bars indicate significant differences among various power levels at the same exposure time. Significant difference was evaluated using One-way ANOVA followed by Tukey’s multiple comparison test (*p* < 0.05).

To evaluate the effects of microwave treatment on physical properties of paddy with increasing exposure time, this study measured key parameters including moisture content, water activity, percentage of grain breakage, and germination rate under different exposure durations. The results showed that at a power of 350 W, the moisture content of paddy gradually decreased as exposure time increased from 10 to 60 s (Figure 3A). Similar decreasing trends in moisture content were also observed at 490 W and 700 W power levels under same exposure durations. Notably, the moisture loss exceeded 1% for paddy samples treated at 490 W and 700 W for an exposure time of 60 s, whereas the effect of the 350 W treatment on moisture loss was relatively weaker. To maintain consistent quality during long-term storage, it is necessary to reduce moisture content of food grains to a wet basis moisture level of ≤14% [36]. In this study, the moisture content of paddy samples treated with microwave was lower than that of the untreated samples. This result implies that microwave heating might enhance the storage quality of the grain without causing excessive drying. As expected with a decrease in moisture content, the water activity of paddy samples also reduced (Figure 3B). Following a 60 s exposure, the water activity decreased from an initial value of 0.69 to 0.63, 0.59, and 0.59 at power levels of 350 W, 490 W, and 700 W, respectively.

The percentage of grain breakage is a key factor affecting the cooking quality of rice [20]. Therefore, the effects of microwave treatment on the breakage of paddy rice were also studied. As shown in Figure 3C, treating paddy at three microwave power levels (350 W, 490 W, and 700 W) for 10 to 60 s did not lead to a significant increase in the grain breakage percentage. These findings indicate that within the 60 s microwave treatment period, microwaves at all three power levels had a minimal impact on the integrity of the paddy kernels, effectively preventing excessive breakage and thereby preserving paddy quality and the feasibility of subsequent processing.

The effect of microwave power levels and exposure times on the germination characteristics of paddy were examined. The results are given in Figure 3D. The germination rate of paddy was 96% before microwave heating. The three microwave power levels (350 W, 490 W, and 700 W) all inhibited the germination rate of paddy seeds, and this inhibitory effect was found to enhance with the increase in exposure time. When the exposure time was extended to 50 and 60 s, the inhibitory effect of 700 W power level on the germination rate became more pronounced, and significant differences in germination rate emerged among the different power levels. These findings demonstrate that exposure of paddy to microwave radiation results in a reduction in germination rate, the extent of which is dependent on both the power level and exposure duration.

### 3.4. Effect of Microwave Treatment on the Total Fungal Load in Paddy

**Figure 4:**
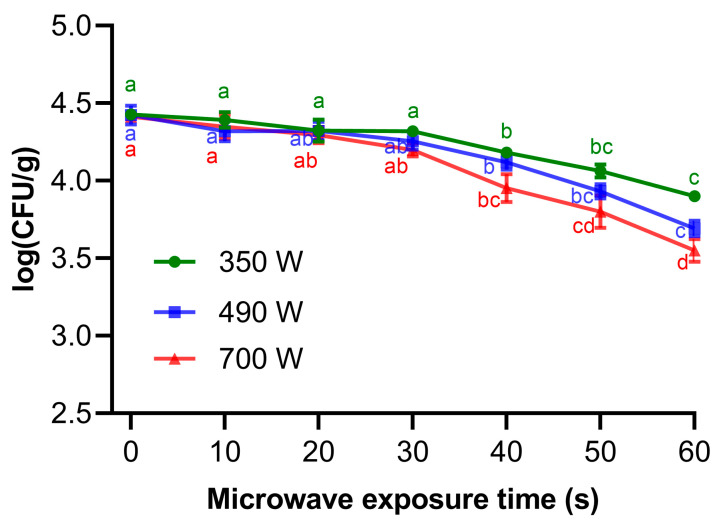
Effect of microwave treatment on total fungal load (log CFU/g) in paddy. Data are presented as mean ± SEM of triplicate samples. The different letters indicate significant differences between different exposure time under the same power level at *p* < 0.05, as determined by One-way ANOVA followed by Tukey’s multiple comparison test.

In this study, we estimated the changes in total fungal counts when 100 g of paddy grains were subjected to microwave treatment at three power levels (350 W, 490 W, and 700 W) for different time intervals (10 s, 20 s, 30 s, 40 s, 50 s, and 60 s). Figure 4 illustrates the impact of microwave treatment on the total fungal load of paddy samples. The logarithmic fungal load of the control group samples was 4.41, which was significantly higher than that of all microwave-treated groups. At the power levels of 350 W, 490 W, and 700 W, the logarithmic fungal load decreased significantly with increasing treatment time and reached the lowest values at 60 s of treatment, which were 3.90 log CFU/g at 350 W, 3.69 log CFU/g at 490 W, and 3.55 log CFU/g at 700 W, respectively. These results indicate that microwave heating can significantly inhibit fungal presence in paddy. Moreover, the highest fungal presence was observed in the untreated samples, while fungal growth decreased gradually with increasing microwave power and exposure time. These findings suggest that the effect of microwave on fungal load and colony formation is dose dependent.

### 3.5. Recovery Percentage of Adults of Three Pest Insects in Paddy After Microwave Exposure

**Figure 5:**
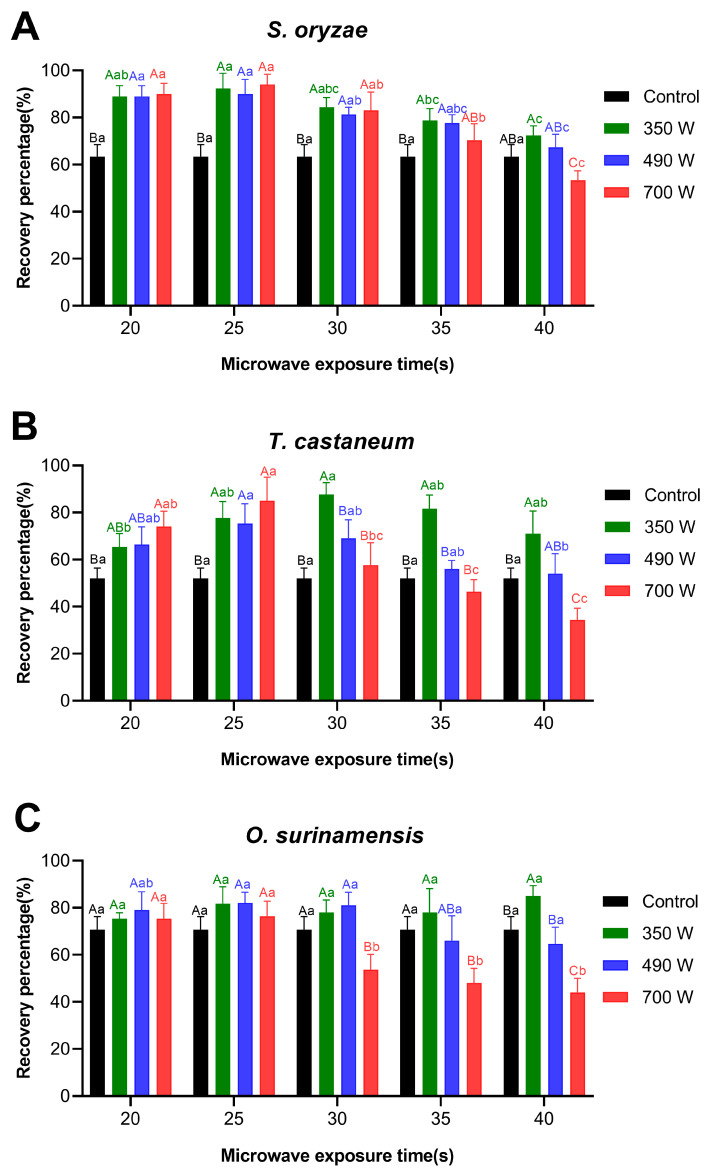
Effect of microwave exposure on the recovery percentage of three stored product insects in paddy. The recovery percentage of *Sitophilus oryzae* (**A**), *Tribolium castaneum* (**B**), and *Oryzaephilus surinamensis* (**C**) after microwave exposure. Data were shown as mean ± SEM. The lowercase letters above the error bars denote significant differences in exposure time under the same power level, and the uppercase letters indicate significant differences between different power levels at the same exposure time. Significant differences among various levels under the same treatment factors were assessed using One-way ANOVA followed by Tukey’s multiple comparison test. Values with different uppercase or lowercase letters are significantly different at *p* < 0.05.

Three species of stored product insect pests were recovered from paddy after microwave treatment and sieving, with the corresponding recovery percentages shown in Figure 5. The results indicated that the three species of stored product insects exhibited different recovery rates under the same microwave treatment conditions. For *S. oryzae*, the recovery percentage following microwave treatment was remarkably higher than in the control group, reaching around 90%. Notably, after exposure times of 20 s, 25 s, and 30 s, the recovery percentage significantly increased under all three microwave power levels, reaching a maximum of 94% for average after 25 s of treatment at 700 W. However, when the exposure time was extended to 35 s, the recovery percentage at 350 W and 490 W treatments showed no significant difference compared to the control group. However, at 700 W, the recovery percentage after 40 s of exposure (the lowest 53.3%) was significantly lower than in the control group.

Under the power of 350 W, microwave exposure for 25 to 40 s significantly increased the recovery percentage of *T. castaneum*, with the highest rate of 87.7% ± 2.5% observed at 30 s. At power levels of 490 W and 700 W, short exposures of 20 and 25 s also enhanced significantly the recovery percentage. Under the power condition of 350 W, microwave exposure for 20 to 40 s could increase the recovery rate of *T. castaneum*, having reached the highest rate of 87.7% ± 2.5% observed at 30 s of exposure. At the power levels of 490 W and 700 W, short exposure times of 20 s and 25 s could also enhance its recovery rate; particularly at 25 s, significant differences were observed compared with the control group under all three power levels. However, when the exposure time was extended to 30, 35, or 40 s at 490 W, the recovery rate showed no significant difference compared to the control group. In contrast, under 700 W, longer exposure durations (35 s and 40 s) resulted in recovery percentages lower than those of the control group. Especially under 700 W, the recovery percentage after 40 s of exposure declined significantly to 33.3% ± 1.9% for 40 s exposure. This may be accounted for by the observation that some of the dead *T. castaneum* were difficult to remove by sieving under these conditions, thereby affecting the statistical results of the recovery percentage.

Under different microwave power levels, the recovery percentage of *O. surinamensis* was as follows: at 350 W, only 40 s of exposure significantly increased the recovery percentage (reached the highest 85% ± 2.5%), while exposure from 20 to 35 s had no significant effect on the recovery percentage. At 490 W, none of the exposures ranging from 20 to 40 s resulted in a significant change in the recovery percentage. At 700 W, no significant effect on the recovery percentage was observed with short exposure times (20 or 25 s). However, when the exposure was extended to 30, 35, or 40 s, a markedly reduced recovery percentage was recorded compared to the control group. This decline may also be attributed to the difficulty in sieving out some dead insects under these experimental conditions, resulting in a lower calculated recovery percentage.

These results indicate that short exposure times and low microwave power (20 or 25 s at 350 W) increased the recovery percentage of *S. oryzae* and *T. castaneum*. This confirms the usefulness of short application times of a low-power microwave oven in assisting the detection of pest insects in stored products. However, microwave treatment did not enhance presence detection of *O. surinamensis* adults, indicating that the efficiency of microwave-assisted detection significantly depends on insect species. This species-specific effect indicates that a single set of microwave parameters cannot be universally applied for the simultaneous detection of all pests. It is difficult to optimize the detection of all three pests simultaneously by using the same microwave parameter. Consequently, when applying microwave heating technology to detect stored product insects, the treatment parameters should be optimized according to the target pest species to significantly enhance the accuracy and applicability of the detection system. It may be best to use a series of treatments tailored to the pest species identified.

## 4. Discussion

During the microwave treatment process, insects are effectively killed when the temperature of the grain medium rapidly rises above the lethal temperature of the insects [33]. Microwave treatment exhibits significant variations in efficacy against different insect species and their developmental stages. A study on microwave treatment of cowpea weevil *Callosobruchus maculatus* showed that eggs were the most susceptible to microwave exposure [37]. When treated at a power exceeding 400 W for 30 or 40 s, complete egg mortality is achieved. Larvae could be entirely killed after exposure for over 30 s at 400 W or for more than 20 s at 800 W. Although the pupae are relatively more tolerant to microwaves, they can still be completely killed when treated at 600 W for 40 s or at 800 W for over 30 s. A similar pattern is observed in *T. castaneum*, where eggs are the most vulnerable to microwave energy, while adults are the most resistant [17,18,38]. *Sitophilus oryzae* infesting rice grains also follows this trend, with eggs being more sensitive to microwave heating than adults [33]. Specifically, both eggs and adults of *S. oryzae* are killed when the temperature of the rice exceeds 55 °C [33]. These findings indicate that, although the lethal effects of microwave treatment on insects differ among developmental stages, it is effective against all stages. Therefore, in practical pest control, strategies should be developed targeting the most tolerant stage to achieve comprehensive control. In this study, we evaluated the lethal effects of microwave treatment on the adults of three common stored product insects in paddy (*S. oryzae*, *T. castaneum* and *O. surinamensis*), aiming to provide a basis for optimizing microwave-based pest control techniques.

The insecticidal efficacy of microwave treatment depends on several factors, such as moisture content in treated cereals and pulses, exposure time, and power [39]. This study has demonstrated that microwave exposure time and power are key factors determining the mortality rate of the three stored product pests, both showing a significant positive correlation with mortality rate. This is consistent with the recent findings of Saheb Abed et al. (2023), who reported that prolonged microwave exposure time effectively increased the mortality of *S. oryzae*, *Sitophilus granarius*, and *Rhyzopertha dominica* [14]. Similarly, it was also found that the mortality rates of stored insects (*T. castaneum*, *C. maculatus* and *S. granarius*) increased with higher microwave power and longer exposure time [40]. This can be attributed to the increased frequency of water molecules in the body of treated insects and the potential impact of microwave radiation on insect enzymes, which can increase membrane fluidity and partially unfold protein molecules, leading to death [20,41]. Furthermore, this study revealed significant differences in susceptibility to the same microwave treatment among the different insect species. Under the same microwave energy, the mortality of *O. surinamensis* was lower than that of the larger *S. oryzae* and *T. castaneum*. This discrepancy may be attributed to one or a combination of the following factors: (i) *O. surinamensis* may have a lower probability of directly absorbing microwave energy, resulting in slower internal heat accumulation; (ii) this species might migrate to cooler zones within the paddy, thereby avoiding exposure to lethal temperatures and reducing its mortality [17,35].

The non-uniform temperature distribution in food materials during microwave heating has been documented in the literature [14,17,35]. This study similarly observed such inhomogeneity, with the central region of paddy samples exhibiting higher surface temperatures compared to the outer areas (Figure 2A). This non-uniformity of microwave heating may be a major problem in grain pest control [5,39]. Specifically, the non-uniformity of microwave heating leads to the emergence of hot and cold spots, between which considerable temperature differences exist [17,35]. In the hot spots, grains may be damaged by high temperatures, thereby affecting germination (a phenomenon observed in this study), while in the cold spots, insects may survive [29]. Our findings indicate that although higher power and prolonged microwave exposure could completely kill *S. oryzae* and *T. castaneum*, they failed to kill all *O. surinamensis*, suggesting that the latter may have survived in cool spots. However, extending the treatment duration to achieve complete insect mortality could severely compromise grain quality. For instance, our results demonstrated that prolonged microwave heating significantly reduced the germination rate of paddy (Figure 3D), which is likely attributed to enzyme denaturation. As also established in a previous study, microwave heating effectively inactivates enzymes, with higher temperatures and longer durations resulting in a greater degree of inactivation [42]. On the other hand, some studies have suggested that some microwave treatment, particularly at low power and short duration, may serve as an effective emerging method for improving the physicochemical and nutritional properties of sorghum grains [26,31]. A similar trend was observed in our study. Moderate microwave treatment resulted in good stability of paddy physical properties, such as moisture content loss, water activity, and grain breakage percentage. Compared with untreated paddy samples, microwave-treated samples showed lower moisture content and water activity, while no significant difference was detected in grain breakage percentage (Figure 3C). In summary, achieving effective pest control using the thermal effect of microwaves relies on higher temperatures, which, however, will lead to a corresponding decrease in the germination rate of grain and moisture content. Therefore, in practical applications, appropriate microwave treatment strategies need to be formulated based on the final use of the grain.

In addition to significantly killing insects in grains, microwave heating also can effectively suppress fungal growth. Our results found that microwave treatment caused a reduction in the total fungal load in paddy. The inhibition of fungal growth after microwave treatment may be due to fungal cell damage caused by heating [26], as fungal cells are highly sensitive to the heat and electromagnetic radiation generated by microwaves. This sensitivity is particularly evident at longer microwave exposure times. In this experiment, the microwave treatment duration reached 60 s, the maximum surface temperatures of paddy samples were 52.5 °C at 350 W, 62.0 °C at 490 W, and 66.3 °C at 700 W (Figure 2B). These high temperatures likely caused irreversible damage to the fungal cells, thereby inhibiting their growth and reproduction. These results are consistent with the findings reported by Almaiman et al. (2021), who reported that microwave treatment can reduce fungal load in sorghum grains [26]. They also found that fungal growth and colony formation are dependent on the microwave power and exposure time, particularly at the longest application time, 45 s, where the surface temperatures reached were 67.4 °C and 72.6 °C for 350 and 500 W [26]. In addition, Shashikumar et al. (2025) investigated the reduction in fungi load on black rice and soft rice using microwave power levels of 1600 W and 1800 W [20]. For both power levels, the total fungal count decreased significantly as exposure time increased in both black rice and soft rice. Overall, this inhibitory effect on fungal growth demonstrates that microwave heating is an effective method for inactivating fungal cells in grains, which is expected to extend their shelf life.

Previous studies have shown that microwave treatment within a specific power range can influence the behavior of stored product insects, causing them to migrate toward the grain surface layer [29,41]. Based on this, sublethal microwave treatment can be employed to assist in screening the number of insects in grain storage. In this study, microwave treatment was applied to paddy samples infested with pests. The results showed that microwave exposure (20 and 25 s) enhanced the recovery percentages of *S. oryzae* and *T. castaneum* in paddy samples. These findings are similar to those reported by Jian et al. [27,29]. We also confirmed that microwave-assisted treatment could improve the detection efficiency of stored product insects. The improved detection efficiency may be attributed to the temperature increase caused by microwave heating, which enhances the activity of *S. oryzae* and *T. castaneum* adults, making it easier to separate and detect. This interpretation is consistent with existing behavioral observations, where researchers directly observed the highly active behavior of *S. oryzae* adults under elevated temperatures during microwave-assisted separation [27]. Additionally, insect species significantly influenced the recovery percentage of pests after microwave treatment [27]. The results of this study showed that although microwave treatment increased the recovery percentages of *S. oryzae* and *T. castaneum*, it did not enhance that of *O. surinamensis*. The exact reason for this observation remains unclear. It is worth noting that previous research reported a significant improvement in the recovery percentage of *O. surinamensis* following microwave treatment [27], which is inconsistent with our results. This discrepancy may be attributed to differences in experimental conditions, such as the maximum exposure duration of 40 s in this study compared to the 150 s treatment applied in earlier research [27].

Beyond exposure duration, other factors could contribute to differences in experimental results. These may include the specific paddy varieties used, the geographical source or strain of the insects, and other uncontrolled or unreported experimental variables. The efficacy of microwave treatment is generally closely influenced by one or a combination of parameters, including microwave power, treatment time, material moisture content, and rice varieties [20,25].

Notably, there exists an inherent trade-off between the microwave parameters required to improve pest detection efficiency and those needed to achieve a high mortality rate. As shown in Figure 5, the treatment conditions that can significantly enhance the detection rates of *S. oryzae* and *T. castaneum* (e.g., 350 W for 20–30 s) only result in partial mortality (Table 1). Conversely, high-power and long-duration treatments that achieve complete mortality (700 W for 60 s) are no longer suitable for detection purposes and will have a negative impact on paddy germination rate (Figure 3D). This trade-off dictates that in the practical application of microwave technology, differentiated strategies must be selected based on the core objective (rapid detection or complete insect pest eradication).

While this study provides essential laboratory-scale parameters, translating these findings to large-scale commercial storage requires further consideration. Factors such as the penetration depth of microwaves in larger grain bulks, the design of continuous or batch processing systems for uniform heating, and the overall energy economics would be critical for practical implementation. Future research should focus on pilot-scale validation to bridge the gap between controlled experiments and industrial application.

## 5. Conclusions

In summary, microwave treatment exhibited good insecticidal potential against *S. oryzae* and *T. castaneum*, and effectively inhibited fungi growth. However, it showed weak efficacy against *O. surinamensis*, and a contradiction existed between the insecticidal efficacy and the grain germination rate. This study revealed the value of microwave treatment in both partial pest control and auxiliary improvement of detection efficiency for specific pests. Nevertheless, its efficacy showed significant interspecific differences, and a parameter trade-off existed among the objectives of insecticidal activity, pest detection, and grain quality preservation. Therefore, future applications require precise optimization of parameters based on target pests and storage scenarios (e.g., seed grain or commodity grain), or integration of microwave treatment into integrated pest management strategies, rather than using it as a single universal solution.

## Figures and Tables

**Table 1:** Immediate and delayed mortality of three stored product insects after microwave exposure in paddy.

Species	Exposure Time (s)	Mortality (%)					
		350 W		490 W		700 W	
		Immediate *	Delayed	Immediate	Delayed	Immediate	Delayed
*S. oryzae*	10	12.00 ± 1.16 Bd	12.00 ± 1.16 Bd	17.33 ± 1.76 ABd	17.33 ± 1.76 ABd	20.00 ± 1.16 Ae	20.00 ± 1.16 Af
	20	32.00 ± 4.16 Ac	37.33 ± 3.33 Ac	34.00 ± 3.06 Ac	40.00 ± 2.31 Ac	40.67 ± 2.40 Ad	46.67 ± 1.76 Ae
	30	36.00 ± 3.45 Cc	42.00 ± 1.16 Cc	54.00 ± 4.00 ABb	59.33 ± 2.91 ABb	56.00 ± 4.16 Ac	62.67 ± 1.33 Ad
	40	62.00 ± 1.15 Ab	66.67 ± 1.76 Ab	65.33 ± 3.53 Ab	69.33 ± 2.91 Ab	72.00 ± 3.06 Ab	73.33 ± 1.76 Ac
	50	72.00 ± 2.00 Cab	73.33 ± 1.76 Cab	80.67 ± 0.67 Ba	82.67 ± 1.76 Ba	89.33 ± 1.76 Aa	90.67 ± 0.67 Ab
	60	79.33 ± 1.76 Ca	80.67 ± 2.40 Ba	88.00 ± 2.31 Ba	88.67 ± 2.91 Ba	100.00 ± 0.00 Aa	100.00 ± 0.00 Aa
*T. castaneum*	10	9.33 ± 1.76 Bd	9.33 ± 1.76 Bf	12.00 ± 1.16 ABd	12.67 ± 1.76 ABe	18.00 ± 1.16 Af	18.67 ± 0.67 Af
	20	26.67 ± 1.76 Ac	28.00 ± 1.16 Ae	28.00 ± 2.31 Ac	29.33 ± 3.33 Ad	31.33 ± 1.33 Ae	34.67 ± 0.67 Ae
	30	30.00 ± 3.46 Bc	40.00 ± 2.31 Bd	35.33 ± 2.91 Bc	41.33 ± 2.67 Bc	53.33 ± 1.76 Ad	54.67 ± 0.67 Ad
	40	58.67 ± 4.67 Ab	63.33 ± 3.33 Ac	66.00 ± 4.16 Ab	66.00 ± 2.00 Ab	66.67 ± 1.76 Ac	70.00 ± 2.31 Ac
	50	64.67 ± 1.76 Cb	67.33 ± 2.40 bBc	76.00 ± 2.00 Bab	76.67 ± 2.40 ABab	87.33 ± 3.53 Ab	88.00 ± 3.06 Ab
	60	78.00 ± 1.16 Ca	80.00 ± 2.31 Ba	84.67 ± 1.76 Ba	86.00 ± 2.31 Ba	99.33 ± 0.67 Aa	100.00 ± 0.00 Aa
*O. surinamensis*	10	6.67 ± 1.76 Ad	8.00 ± 2.00 Ad	10.00 ± 1.16 Ac	12.00 ± 1.16 Ad	12.00 ± 2.31 Ac	13.33 ± 2.40 Ad
	20	14.67 ± 2.40 Acd	17.33 ± 1.33 Acd	20.00 ± 2.31 Ac	22.67 ± 1.33 Acd	26.00 ± 4.16 Abc	26.67 ± 4.06 Acd
	30	28.67 ± 3.71 Ac	29.33 ± 3.52 Ac	36.67 ± 4.06 Ab	38.67 ± 4.67 Ab	42.00 ± 4.16 Ab	44.00 ± 3.06 Ac
	40	51.33 ± 2.91 Ab	52.67 ± 3.52 Ab	58.67 ± 3.53 Aa	60.67 ± 3.33 Aa	65.33 ± 5.81 Aa	66.67 ± 5.21 Ab
	50	62.67 ± 2.91 Aab	64.67 ± 2.91 Aab	66.67 ± 2.40 Aa	68.67 ± 0.67 Aa	70.67 ± 3.53 Aa	72.00 ± 4.62 Aab
	60	69.33 ± 4.06 Aa	72.00 ± 2.31 Aa	72.00 ± 4.16 Aa	74.67 ± 4.67 Aa	82.00 ± 3.06 Aa	86.00 ± 2.31 Aa

* Immediate and delayed mortality were shown as mean ± SEM from three replicates. Significant differences among various levels under the same treatment factors were evaluated using One-way ANOVA followed by Tukey’s multiple comparison test, and were denoted by different lowercase letters (for comparisons across exposure times within the same power level) or uppercase letters (for comparisons across power levels within the same exposure time).

## Data Availability

The original contributions presented in this study are included in the article. Further inquiries can be directed to the corresponding authors.
